# Data on distribution of heat transfer coefficient and profiles of velocity and turbulent characteristics behind a rib in pulsating flows

**DOI:** 10.1016/j.dib.2020.106485

**Published:** 2020-11-02

**Authors:** Irek Davletshin, Andrey Mikheev, Nikolay Mikheev, Radif Shakirov

**Affiliations:** Institute of Power Engineering and Advanced Technologies, FRC Kazan Scientific Center, Russian Academy of Sciences, 2/31 Lobachevskogo Str., Kazan 420111, Russia

**Keywords:** Flow separation, Pulsating flow, Forcing frequency, Forcing amplitude, Heat transfer coefficient, Flow structure, Turbulent characteristics

## Abstract

The paper presents experimental data on heat transfer and kinematic structure of steady and pulsating flows behind a rib. Several forcing frequencies and one non-dimensional amplitude of pulsation are considered. Distributions of heat transfer coefficient were obtained in the separation region. Optical measurements yielded the profiles of velocity and turbulent characteristics of flow at representative coordinates of the separation region.

## Specifications Table

**Table:** 

Subject	Mechanical Engineering
Specific subject area	Hydrodynamics and Thermophysics (focusing on the effect of flow pulsations on heat transfer and kinematic structure of separated flows)
Type of data	Tables Figures Text File
How data were acquired	Heat transfer measurements, optical measurements of velocity fields
Data format	Raw Analyzed Filtered
Parameters for data collection	Heat transfer study: *f*, Hz, β=*A*_U_/*U*_0_*Q*, m^3^/h *U*_0_=*Q*/*F*_0_, m/s *Re*=*U*_0_*e*/ν Steady - 10 6 - 395 1.7 – 6.36 3400 - 12,700 5 - 35 0.5 105 - 396 1.69 – 6.38 3380 - 12,760 Investigation of hydrodynamics: *f*, Hz β=*A*_U_/*U*_0_*Q*, m^3^/h *U*_0_=*Q*/*F*_0_, m/s *Re*=*U*_0_*e*/ν Steady - 106 1.7 3400 10 0.5 105 1.69 3380 Here: *f* – forcing frequency; β – non-dimensional forcing amplitude; *Q* – volumetric air flow rate; *U*_0_ – bulk velocity.
Description of data collection	Data were collected using a special experimental setup. Optical method SIV (Smoke Image Velocimetry) was employed for the research into hydrodynamics. Heat transfer was measured by a heated printed circuit board with copper tracks that simultaneously served as resistance thermometers. Heat transfer coefficients were estimated from heat release values and temperature difference between the wall and the flow.
Data source location	Institution: Institute of Power Engineering and Advanced Technologies, FRC Kazan Scientific Center, Russian Academy of Sciences City/Town/Region: Kazan Country: Russia
Data accessibility	https://data.mendeley.com/datasets/cmcwv9x39z/1
Related research article	I.A. Davletshin, A.N. Mikheev, N.I. Mikheev, R.R. Shakirov, Heat transfer and structure of pulsating flow behind a rib, International Journal of Heat and Mass Transfer 160 (2020) 120173 https://doi.org/10.1016/j.ijheatmasstransfer.2020.120173

## Value of the Data

• The obtained combination of experimental data on heat transfer and kinematic structure of separated flows subjected to forced pulsations will benefit the search for the correlation between the heat transfer and hydrodynamics.
• Main patterns of the influence of forced pulsations on heat transfer in the separation region are revealed. Distributions of heat transfer coefficient are generalized well when the dimensionless frequency (Stanton number) is used.
• Profiles of velocity and turbulent characteristics were obtained at the representative coordinates of the separation region in steady and pulsating flows.
• The research also focuses on the vortex street behind the rib, which is an important component of the kinematic structure of flow. Parameters of this vortex street depend on the frequency and amplitude of pulsations.
• Simultaneous analysis of heat transfer and hydrodynamics of pulsating flows was performed; correlation between local heat transfer and transverse velocity was revealed.

## 1 Data Description

The obtained heat transfer results contain distributions of the heat transfer coefficient over the channel wall behind the rib in steady and pulsating flows (air with ambient parameters). Experimental data were acquired for several flow rates, several frequencies and single amplitude of forced pulsations.

The data on kinematic structure of flow include the profiles of velocity and turbulent characteristics at representative coordinates of the separation region. These data were obtained in much detail for one flow rate. Steady-state flow and pulsating flow at a single one forcing frequency and one level of forced amplitude were considered. Similarity of geometry and flow regimes allowed direct comparison between heat transfer and hydrodynamic parameters.

## 2 Experimental Design, Materials and Methods

### 2.1 Materials

Heat transfer behind a rib in pulsating air flows was investigated using a special experimental setup. The test section was a 1.2 m long rectangular channel with a cross section *F*_0_=0.115 × 0.15 m^2^. An aluminum square rib with the height *e* = 30 mm (rib dimensions: 30 × 30 × 150 mm^3^) was installed on one of the 0.15 m wide walls at the distance of 0.1 m from the inlet. A 455 mm long section for heat transfer measurements was mounted immediately downstream of the rib. Stable flow rate was provided by critical flow nozzles. Close to harmonic pulsation pattern was generated by a rotating flap at the channel outlet.

Transparent walls (glass and polycarbonate) facilitated experimental investigation of kinematic structure of flow. Aerosol was supplied from the preparation chamber to the channel entrance in order to visualize the flow structure. Vector fields of velocity were estimated from the analysis of displacements of turbulent structures visualized by aerosol. The flow patterns were filmed with a high-speed camera in a light sheet illuminating the axial plane of the channel.

### 2.2 Method of investigation

Heat transfer between the wall and the flow was provided by heating the measurement section of the wall by direct current supplied from a battery. A printed circuit board was employed as a heated wall. The tracks of this board simultaneously served as resistance thermometers registering the local wall temperatures. The distributions of heat transfer coefficient behind the rib were derived from convective heat fluxes and difference between the wall and flow temperatures. Convective heat fluxes were estimated based on the heat generated by electrical current taking into account the heat losses estimated from the heat balance equation and heat flux equations (thermal conductivity, radiation, natural convection). Measurement uncertainties were estimated. The method was described and tested in [2].

Velocity fields and Reynolds stresses were measured using the optical method SIV (Smoke Image Velocimetry) based on digital processing of flow pattern videos. SIV estimated vector fields of velocity analyzing the displacements of turbulent structures visualized by aerosol [3], [4], [5].

## 3 Data Obtained

### 3.1 Heat transfer

Flow parameters considered in the heat transfer study are given in Table 1.

**Fig. 1:**
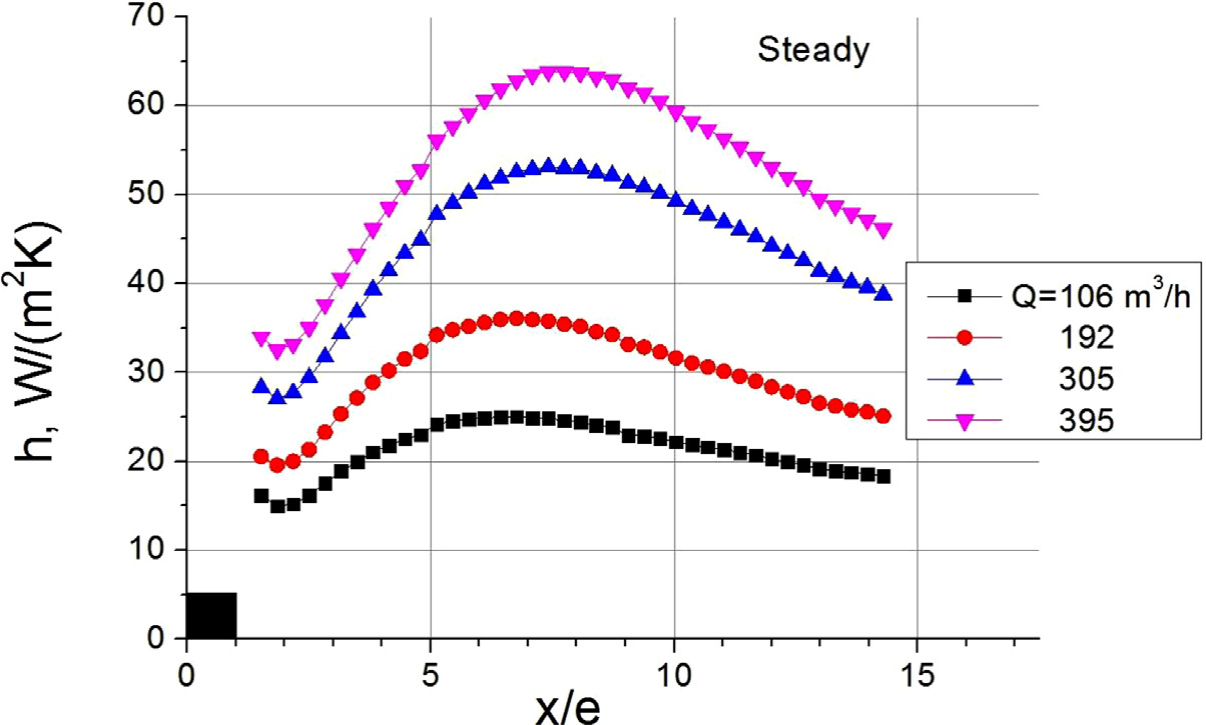
Heat transfer coefficient in the separation region of steady flow.

**Fig. 2:**
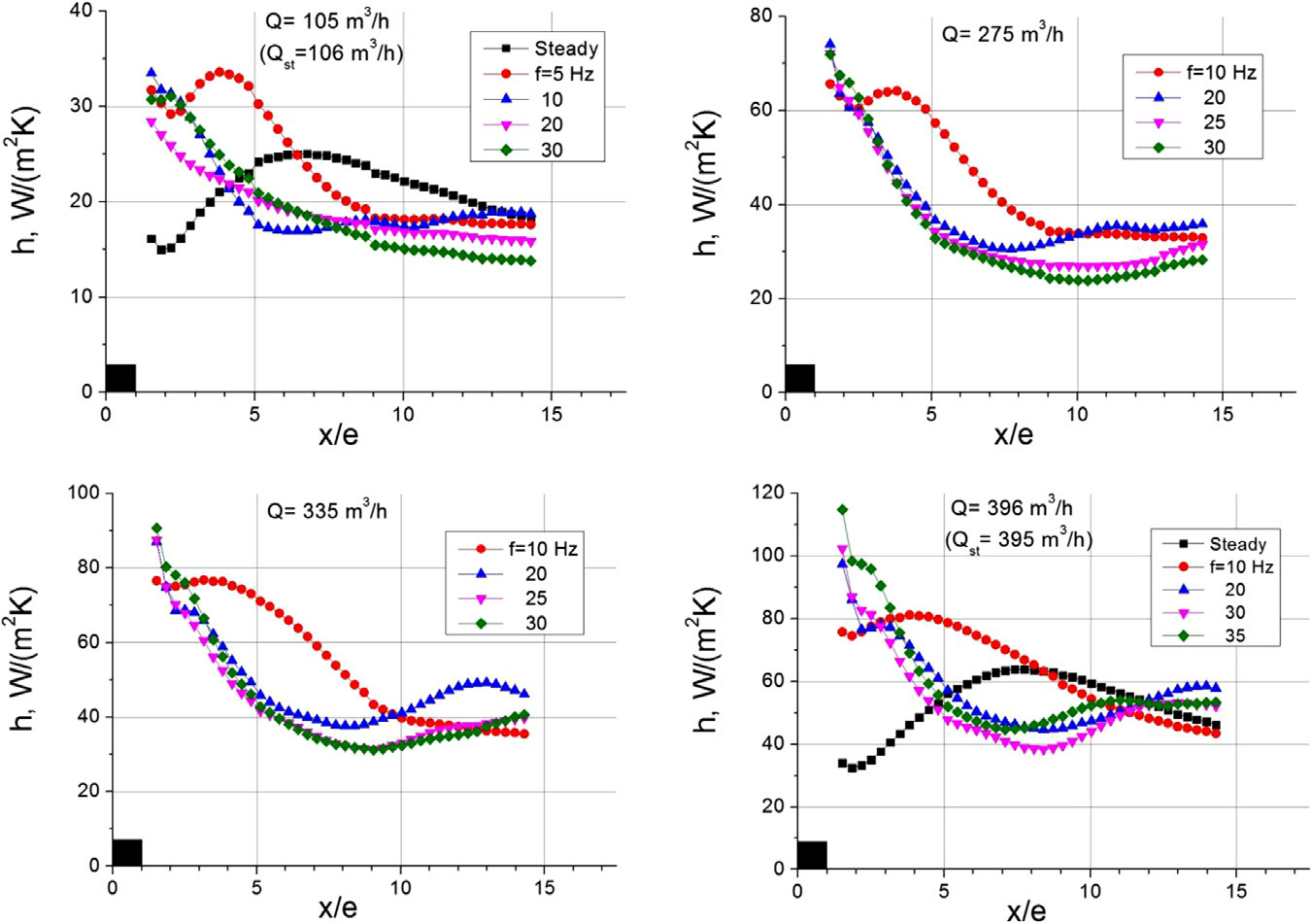
Heat transfer coefficient in the separation region of pulsating flow.

The volumetric air flow rate, *Q*, was provided by the critical flow nozzles. The flow rates brought to standard conditions (20 °C and 1.013•10^5^ Pa) were estimated taking into consideration the temperature and pressure before the nozzles. The bulk velocity, *U*_0_, was calculated for the coordinate located far away from the rib. The experimental data on heat transfer coefficient along the separation region of steady and pulsating flows are given in Fig. 1, Fig. 2, respectively. X-axis in both figures is the streamwise coordinate *x* normalized by the rib height *e*. Y-axis is the heat transfer coefficient *h*. Distributions were obtained in the streamwise axial plane of the channel. Heat transfer in the corners was not considered. For illustrative purposes, the rib is shown as a black square with a proper scale in the figures.

**Table 1:** Regime parameters implemented during heat transfer experiments.

*f*, Hz	β=*A*_U_/*U*_0_	*Q*, m^3^/h	*U*_0_=*Q*/*F*_0_, m/s	*Re*=*U*_0_*e*/ν
Steady	–	106–395	1.7–6.36	3400–12,700
5–35	0.5	105–396	1.69–6.38	3380–12,760

### 3.2 Hydrodynamics

Detailed investigation of hydrodynamics was carried out for steady and pulsating flow cases (Table 2). The flow parameters were measured at *x/e* = 1.05; 2; 3; 4; 5; 6; 8; 10 (*x* is the streamwise coordinate with the origin at the leading edge of the rib).

**Table 2:** Regime parameters implemented during the study of hydrodynamics.

*f*, Hz	β=*A*_U_/*U*_0_	*Q*, m^3^/h	*U*_0_=*Q*/*F*_0_, m/s	*Re*=*U*_0_*e*/ν
Steady	–	106	1.7	3400
10	0.5	105	1,69	3380

**Fig. 3:**
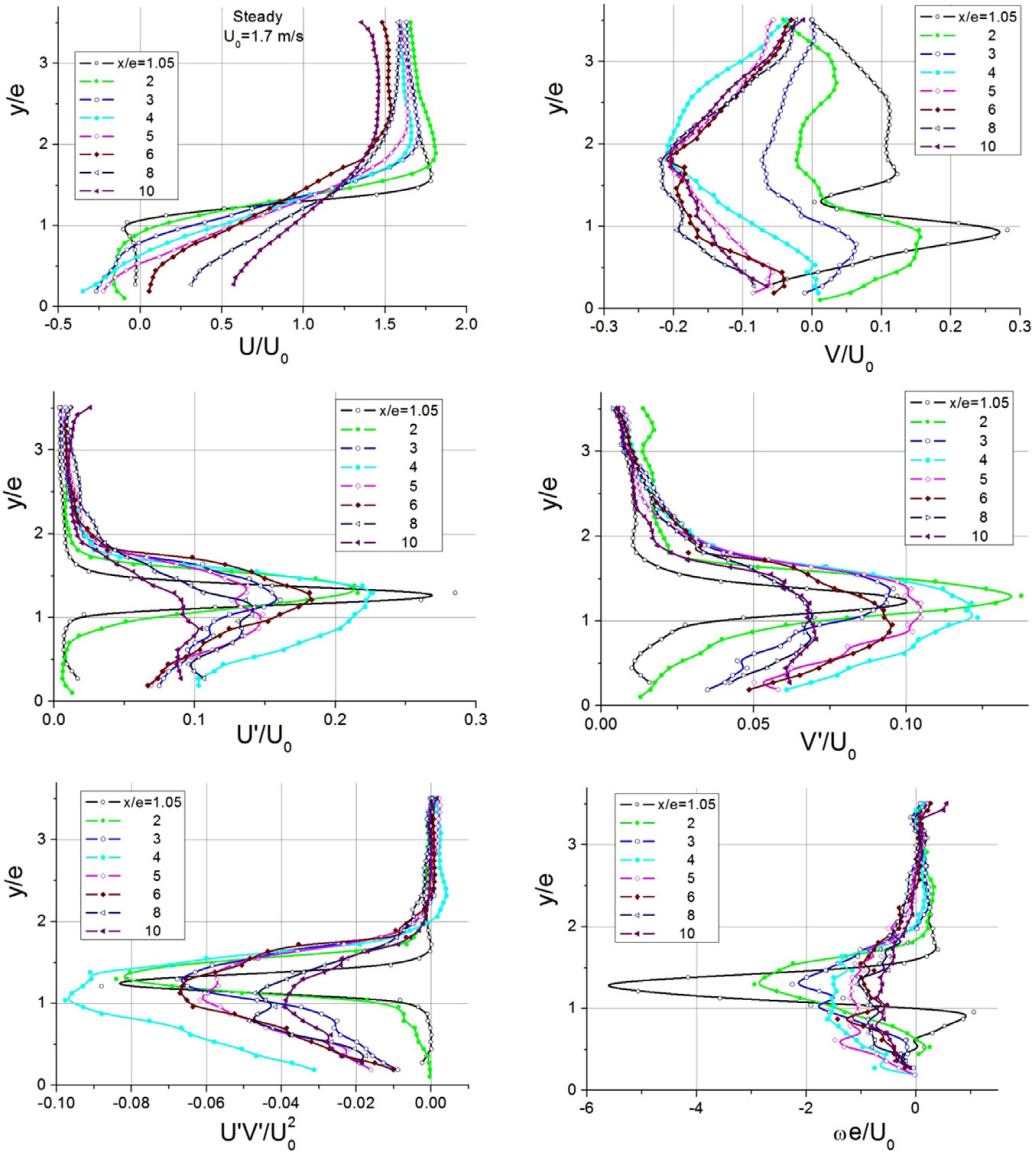
Profiles of hydrodynamic parameters in the separation region of steady flow.

The profiles of velocity and turbulent characteristics in steady flows are presented in Fig. 3, which shows streamwise (*U*) and transverse (*V*) velocities, their turbulent components (*Uʹ* and *Vʹ*), Reynolds stresses (*UʹVʹ*) and vorticity (ω) normalized by bulk velocity and rib height (*e*).

**Fig. 4:**
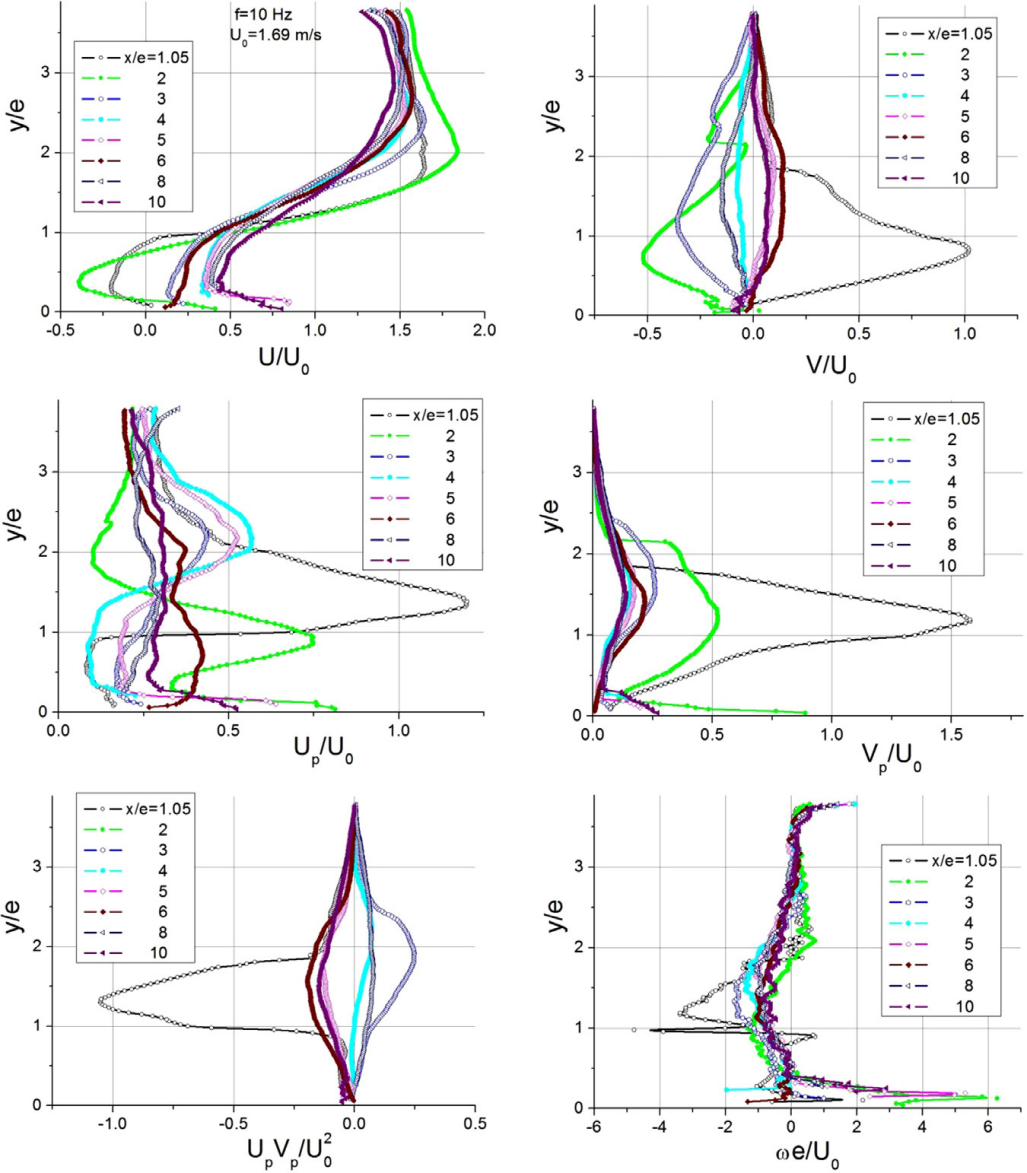
Profiles of hydrodynamic parameters in the separation region of pulsating flows.

Profiles of velocity and fluctuations in pulsating flows are demonstrated in Fig. 4. Similarly, the streamwise (*U*) and transverse (*V*) velocities are plotted. Here, the fluctuations are considered as a combination of periodic and turbulent components of velocity *U_p_=Ũ+Uʹ* and *V_p_=Ṽ+Vʹ*, where *Ũ*=*A*_U_Sin(2π*f*τ) and *Ṽ*=*A*_V_Sin(2π*f*τ).

**Fig. 5:**
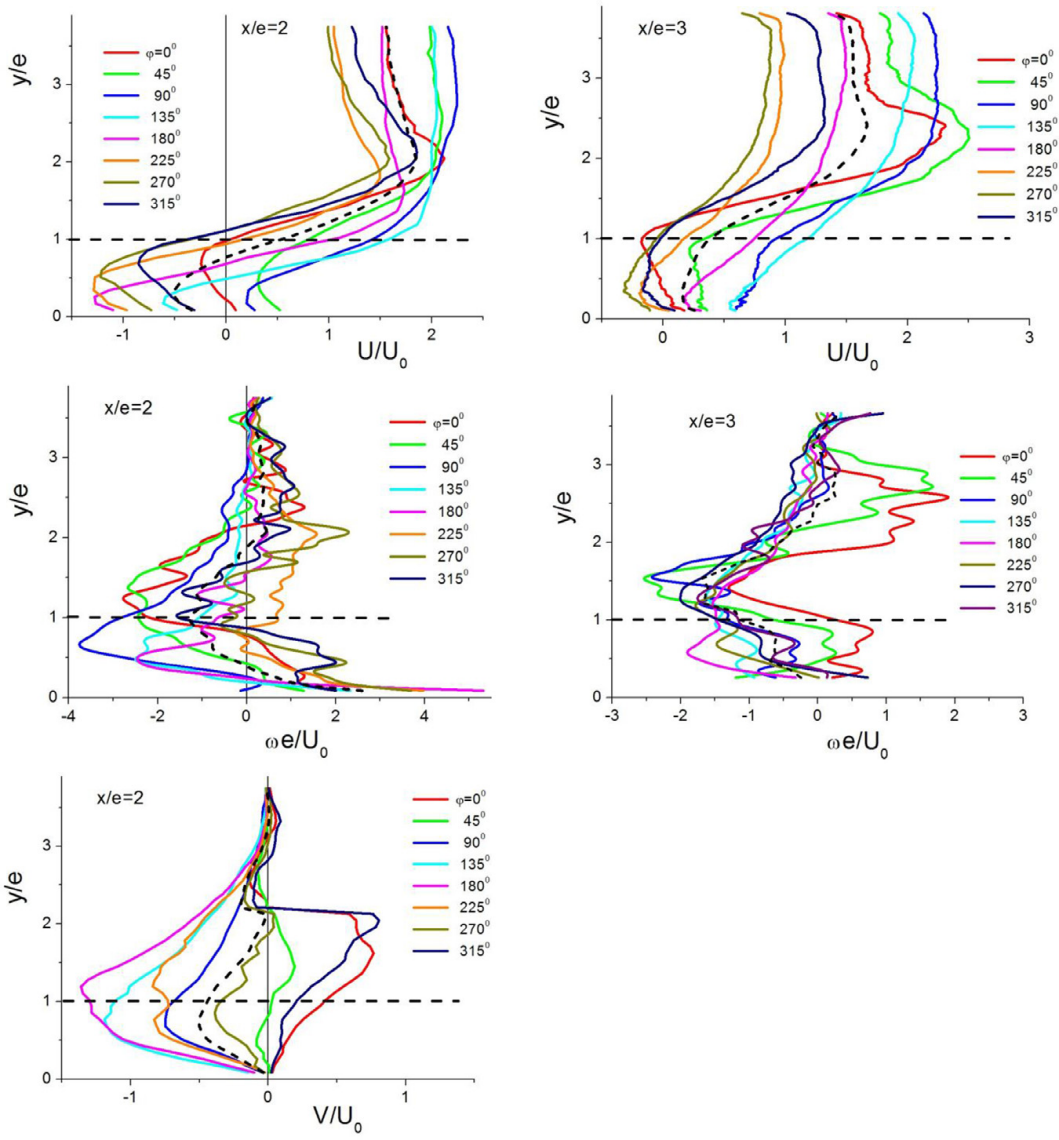
Behavior of hydrodynamic parameters during one period of forced pulsations.

Profiles of flow parameters in different phases φ (φ=2π*f*τ) of forced pulsations were built for two representative coordinates (*x/e* = 2 and *x/e* = 3) with the intention to analyze the evolution of hydrodynamic processes in separated pulsating flows (Fig. 5). Each curve shows a profile in the given phase averaged over a large number of periods. Dashed lines are the profiles of respective parameters averaged over time τ (over phases). Horizontal dashed line marks the rib top.

## 4 Data Analysis

The experimental data on heat transfer and hydrodynamics obtained in the channels of identical geometry at identical regime parameters can be employed for combined analysis of these data and for the search for correlation between these processes. Such an analysis was performed in related research paper [1].

## Ethics Statement

This material is the authors' own original work, which has not been previously published elsewhere

## CRediT authorship contribution statement

**Irek Davletshin:** Formal analysis, Writing - review & editing. **Andrey Mikheev:** Methodology, Writing - review & editing. **Nikolay Mikheev:** Investigation. **Radif Shakirov:** Investigation.

## Declaration of Competing Interest

The authors declare that they have no known competing financial interests or personal relationships that could have appeared to influence the work reported in this paper.
